# Dark-Blood Late Gadolinium Enhancement MRI Is Noninferior to Bright-Blood LGE in Non-Ischemic Cardiomyopathies

**DOI:** 10.3390/diagnostics13091634

**Published:** 2023-05-05

**Authors:** Jan M. Brendel, Robert J. Holtackers, Jan N. Geisel, Jens Kübler, Florian Hagen, Meinrad Gawaz, Konstantin Nikolaou, Simon Greulich, Patrick Krumm

**Affiliations:** 1 Department of Radiology, Diagnostic and Interventional Radiology, University of Tübingen Hoppe-Seyler-Straße 3, 72076 Tübingen, Germany; 2 Department of Radiology and Nuclear Medicine, Maastricht University Medical Centre, 6229 HX Maastricht, The Netherlands; 3 Cardiovascular Research Institute Maastricht (CARIM), Maastricht University, 6229 ER Maastricht, The Netherlands; 4 Department of Internal Medicine III, Cardiology and Angiology, University of Tübingen Otfried-Müller-Straße 10, 72076 Tübingen, Germany

**Keywords:** magnetic resonance imaging, heart, contrast media, gadolinium, LGE, cardiomyopathies, dark blood, bright blood

## Abstract

**(1) Background and Objectives:** Dark-blood late gadolinium enhancement has been shown to be a reliable cardiac magnetic resonance (CMR) method for assessing viability and depicting myocardial scarring in ischemic cardiomyopathy. The aim of this study was to evaluate dark-blood LGE imaging compared with conventional bright-blood LGE for the detection of myocardial scarring in non-ischemic cardiomyopathies. **(2) Materials and Methods:** Patients with suspected non-ischemic cardiomyopathy were prospectively enrolled in this single-centre study from January 2020 to March 2023. All patients underwent 1.5 T CMR with both dark-blood and conventional bright-blood LGE imaging. Corresponding short-axis stacks of both techniques were analysed for the presence, distribution, pattern, and localisation of LGE, as well as the quantitative scar size (%). **(3) Results:** 343 patients (age 44 ± 17 years; 124 women) with suspected non-ischemic cardiomyopathy were examined. LGE was detected in 123 of 343 cases (36%) with excellent inter-reader agreement (κ 0.97–0.99) for both LGE techniques. Dark-blood LGE showed a sensitivity of 99% (CI 98–100), specificity of 99% (CI 98–100), and an accuracy of 99% (CI 99–100) for the detection of non-ischemic scarring. No significant difference in total scar size (%) was observed. Dark-blood imaging with mean 5.35 ± 4.32% enhanced volume of total myocardial volume, bright-blood with 5.24 ± 4.28%, *p* = 0.84. **(4) Conclusions:** Dark-blood LGE imaging is non-inferior to conventional bright-blood LGE imaging in detecting non-ischemic scarring. Therefore, dark-blood LGE imaging may become an equivalent method for the detection of both ischemic and non-ischemic scars.

## 1. Introduction

Late gadolinium enhancement (LGE) is the most established imaging modality for myocardial tissue characterisation in cardiac magnetic resonance (CMR). It is routinely used to assess and quantify myocardial fibrosis, irrespective of an ischemic or non-ischemic origin [1,2,3,4]. The ability to detect areas of scarring within the myocardium using LGE is critical for the diagnosis of patients with cardiomyopathy. Scarring or fibrosis compromises the structural integrity of the myocardium, predisposing to dysfunction leading to heart failure, arrhythmias, and even sudden cardiac death. Therefore, in addition to its great diagnostic value, LGE has been shown in several studies to have a high predictive value, identifying patients at high risk for adverse cardiac events and death [5,6,7,8,9,10]. Various LGE techniques have been developed for the purpose of distinct scar assessment and its delineation from healthy myocardium. Over time, numerous LGE techniques have been introduced, each with the goal of improving myocardial scar detection. In recent years, the so-called dark-blood LGE technique has been proposed to further improve the contrast between subendocardial ischemic scar tissue and the ventricular blood pool [11]. This technique has shown promising results in improving the accuracy and specificity of LGE. Previous studies compared dark-blood LGE with conventional bright-blood LGE in the detection of ischemic scarring, suggesting that dark-blood LGE allows for better delineation and quantification, thus improving the diagnosis of ischemic cardiomyopathy [12,13]. Compared to ischemic scars, non-ischemic scars show different forms of distribution within the myocardium, including linear or patchy types of enhancement. In addition, non-ischemic scars are located mid-wall or subepicardial, typically sparing subendocardial regions supplied by a specific coronary artery. Although some studies have reported on the detection of non-ischemic areas of fibrosis [3,14], there is a lack of data evaluating the diagnostic performance of dark-blood LGE versus conventional bright-blood LGE in visualising non-ischemic scars in a direct head-to-head comparison. To date, there is no clear recommendation for the potential use of dark-blood LGE in cardiac MRI for non-ischemic cardiomyopathy.

We hypothesised that the detection of non-ischemic scars may not differ between the two LGE techniques. Therefore, the aim of this non-inferiority cardiac MRI study was to evaluate the diagnostic performance of dark-blood LGE compared with conventional bright-blood LGE in the detection of non-ischemic myocardial scarring.

## 2. Materials and Methods

### 2.1. Study Population

In this single-centre study (Tübingen University Hospital, Tübingen, Germany), we prospectively enrolled patients referred for cardiac MRI (CMR) between January 2020 and March 2023 for clinical suspicion of non-ischemic cardiomyopathy. Patients with incomplete LGE data sets, insufficient image quality or an ischemic LGE pattern were excluded. The study was approved by the Institutional Ethical Review Board, and all patients provided written, informed consent to participate in the study.

### 2.2. Cardiac MRI Image Acquisition

All patients underwent both conventional bright-blood late gadolinium enhancement and dark-blood late gadolinium enhancement as part of the cardiac MRI imaging protocol using a 1.5 T scanner (MAGNETOM Aera, SIEMENS Healthcare, Erlangen, Germany) within the same scan. In detail, ten minutes after intravenous injection of 0.15 mmol/kg gadobutrol (Gadovist, Bayer Healthcare, Leverkusen, Germany) [15], both LGE techniques were applied according to current recommendations [16]. Dark-blood LGE was performed at 10 min after contrast injection, immediately followed by bright-blood LGE (starting at 15 min after contrast injection). For both LGE methods, an inversion time (TI) scout scan was performed to select the optimal TI. For bright-blood LGE, the TI was set to null viable myocardium of the left ventricle; whereas for dark-blood LGE, the TI was set to null the left ventricular blood pool signal. Sequence parameters for bright-blood LGE were: readout type steady state free precession, echo time 1.24 ms, flip angle 45°, acquired resolution 1.33 × 1.33 mm^2^; phase sensitive inversion recovery (PSIR) steady state free precession, TE 1.26 ms, flip angle 90°, acquired resolution 1.48 × 1.48 mm^2^. For dark-blood LGE, 10–15 2D 8-mm short-axis slices and one 4-chamber slice were acquired; for conventional bright-blood LGE, a 3D data set was acquired and reconstructed in identical slices. The mechanism of the used dark-blood LGE method without additional magnetisation preparation (blood nulled PSIR LGE) has been described in detail previously [17].

### 2.3. Cardiac MRI Image Analysis

Cardiac magnetic resonance image analysis was performed in three readings by readers with different levels of experience: reading 1 by J.M.B. (6 years of cardiac MRI experience), reading 2 by J.N.G. (1 year of cardiac MRI experience), reading 3 in consensus by P.K. (13 years of cardiac MRI experience) and S.G. (22 years of cardiac MRI experience). Analysis was performed according to the recommendations of the Society for Cardiovascular Magnetic Resonance (SCMR) [18,19] using a dedicated commercially available software package (cvi42 version 5.14, Circle Cardiovascular Imaging Inc, Calgary, AB, Canada). Readers were blinded to the clinical data. In all short-axis LGE slices, endocardial and epicardial contours of the left ventricle were manually delineated, followed by an automated calculation of total scar size using a threshold of 2 SD above remote myocardium [18]. LGE distribution (linear or patchy) and pattern (subendocardial, transmural, mid-wall, subepicardial) were noted [5]. LGE localisation was assessed segmentally according to the adapted American Heart Association 16-segment model, excluding the apical segment 17 [20]. Confidence in the presence or absence of scarring was assessed using a 4-point scale (1 = nondiagnostic exam, 2 = low confidence, 3 = moderate confidence, 4 = high confidence). The CMR reporting and diagnosis of “myocarditis”, “non-ischemic cardiomyopathy”, or “normal” was made in accordance with current SCMR recommendations and ESC guidelines [21,22,23].

### 2.4. Statistical Analysis

A priori power calculation was performed, and sample size estimation was based on Tango [24] (alpha = 0.05; power = 0.90; proportion discordant 0.10), the number to be included was *n* = 343 patients. Data distribution was assessed using histograms and measures of skewness and kurtosis. Myocardial scar size (normally distributed) was compared using a paired samples *t*-test (JMP, version 16.2, SAS Institute Inc., Heidelberg, Germany). Differences in myocardial scar size measurements between conventional bright-blood and dark-blood LGE were assessed by Bland–Altman analysis (MedCalc, Version 18.1, MedCalc Software Ltd., Ostend, Belgium). Inter-reader variability of LGE scar size measurements (%) was assessed using intraclass correlations. The diagnostic performance of dark-blood LGE in non-ischemic cardiomyopathy was assessed using the MedCalc diagnostic test evaluation calculator (version 20.112, MedCalc Software Ltd., Ostend, Belgium), considering bright-blood LGE as the reference standard. A two-sided McNemar test was performed to test the marginal homogeneity of both LGE techniques in the dichotomous diagnosis of LGE-positive patients (LGE-positive vs. -negative). Cohen’s κ statistic was used to assess inter-reader agreement for the presence of LGE. Wilcoxon signed rank test for paired samples was used to compare the readers’ confidence scores in assessing the presence or absence of scarring in dark-blood and bright-blood images. Continuous data are presented as mean ± standard deviation. Categorical data are expressed as frequencies (%). *p*-values < 0.05 were considered to indicate a significant difference.

## 3. Results

### 3.1. Patient Characteristics

**Figure 1:**
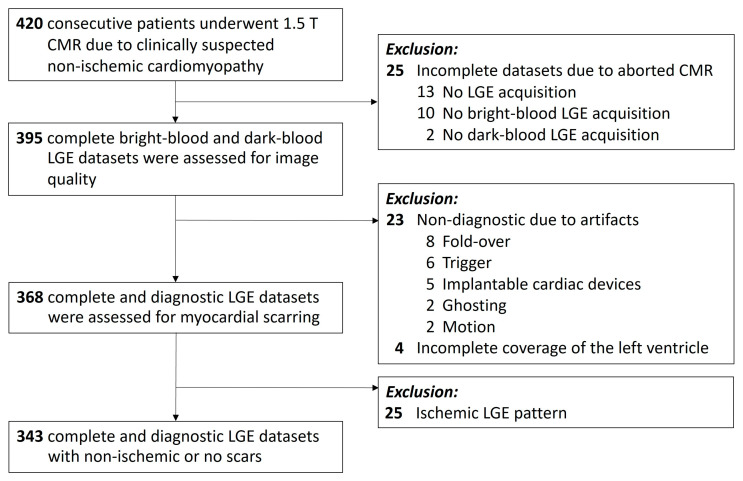
Flowchart showing patient enrolment and reasons for exclusion from the study. Patients referred for cardiac MRI because of clinical suspicion of non-ischemic cardiomyopathy were prospectively enrolled at a single centre (University Hospital of Tübingen) between January 2020 and March 2023. Patients with incomplete LGE data sets, insufficient image quality, or an ischemic LGE pattern were excluded. *CMR =* Cardiac magnetic resonance. *LGE =* Late gadolinium enhancement.

A total of 420 consecutive patients underwent 1.5 T cardiac magnetic resonance (CMR) for clinically suspected non-ischemic cardiomyopathy. A total of 25 datasets were incomplete due to missing acquisition of LGE (*n* = 13), the bright-blood datasets (*n* = 10) or the dark-blood datasets (*n* = 2). Figure 1. 23 cases were excluded because of insufficient image quality due to artifacts: fold-over (*n* = 8), trigger (*n* = 6), MR conditional implantable cardiac devices (*n* = 5), ghosting (*n* = 2), and motion (*n* = 2). *N* = 4 datasets were excluded due to incomplete coverage of the left ventricle. Evaluating the remaining 368 complete and diagnostic datasets, we further excluded cases with ischemic LGE patterns (*n* = 25). Finally, bright-blood and dark-blood LGE data sets from 343 patients (age 44 ± 17 years; age range 18 to 82 years) were used for comparative analysis. The study population consisted of 124/343 (36%) women and 219/343 men (64%).

Baseline characteristics of the study population are shown in Table 1. The most common cardiac MRI diagnosis was myocarditis (*n* = 125/343, 36%), followed by patients without abnormalities on CMR (*n* = 109/343, 32%), and patients with different forms of non-ischemic cardiomyopathy (*n* = 109/343, 32%). In detail: *n* = 31 with hypertrophic cardiomyopathy, *n* = 29 with dilated cardiomyopathy, *n* = 3 with arrhythmogenic cardiomyopathy, *n* = 3 with non-compaction cardiomyopathy, *n* = 3 with tako-tsubo cardiomyopathy, *n* = 2 amyloidosis, *n* = 1 with peripartum cardiomyopathy, and *n* = 37 not further classified non-ischemic cardiomyopathy.

### 3.2. Evaluation of Left Ventricular LGE Frequency, Pattern, and Localisation

**Figure 2:**
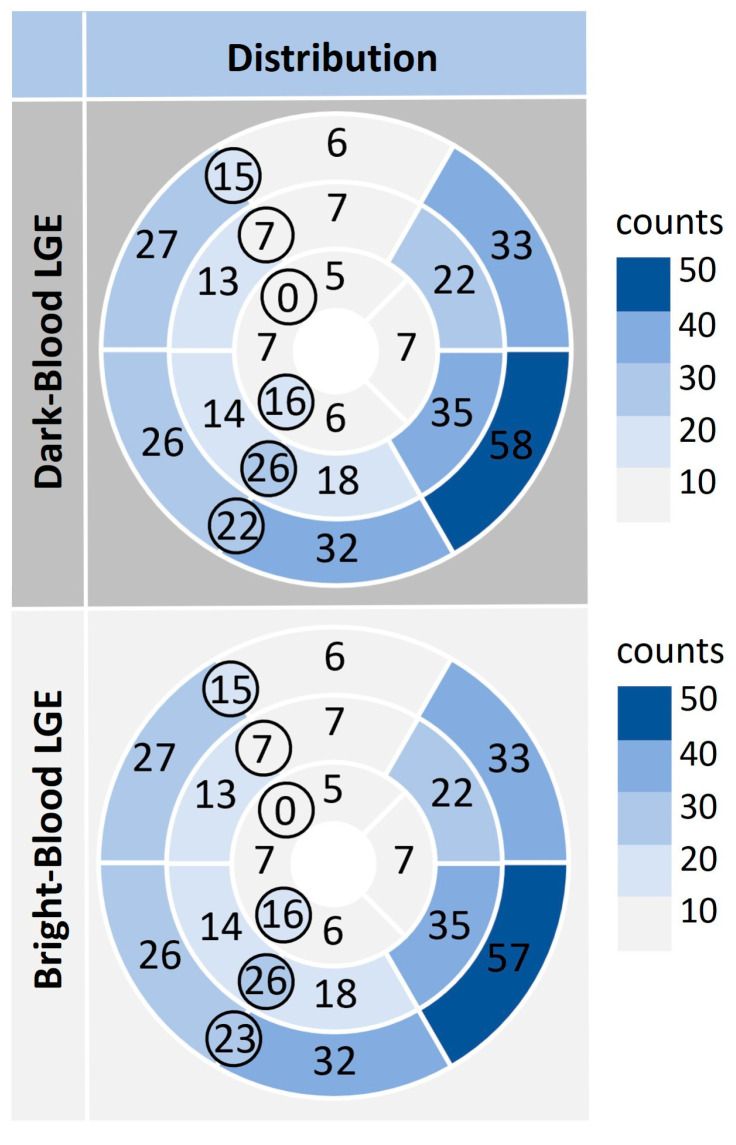
Bullseye plots represent the distribution of late gadolinium enhancement (LGE) depicted by the dark-blood LGE technique (top row) and the bright-blood LGE technique (bottom row). Numbers are the counts of LGE appearances per AHA—segment and at the right ventricular insertion points (circled). The blue colouring serves to better visualise the number of segmental counts (dark blue = many counts, light blue/grey = few counts).

Late gadolinium enhancement was present in 123 of 343 cases (36%). Regarding the distribution of LGE, linear LGE was found in 72 of 123 cases (59%), and 70 of 123 cases (57%) showed patchy LGE. All scars were of a non-ischemic type with a mid-wall LGE pattern (87 of 123 cases, 71%) or a subepicardial LGE pattern (63 of 123 cases, 51%). LGE was predominantly located in the basal inferolateral wall. The inferior right ventricular insertion points showed LGE more frequently than the anterior right ventricular insertion points. The localisation of LGE with segmental counts is depicted in Figure 2.

### 3.3. Comparison of Total Scar Size

**Figure 3:**
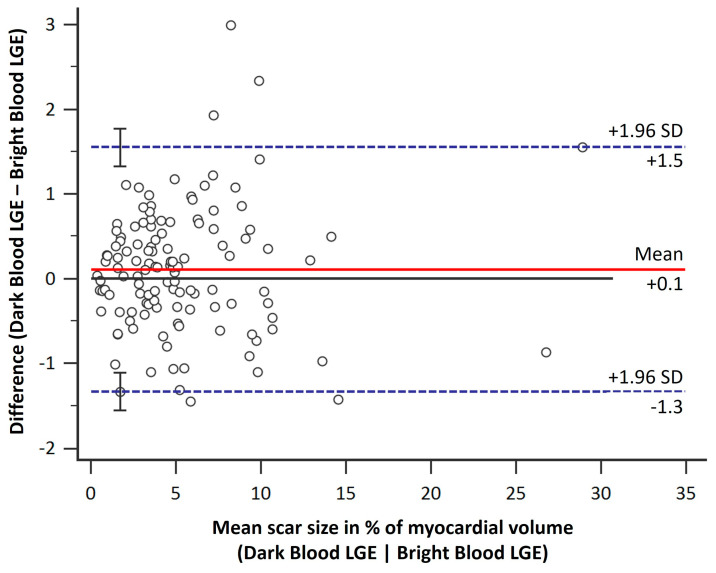
Bland–Altman plot of myocardial scar size as portrayed by dark-blood late gadolinium enhancement (LGE) and bright-blood LGE. No significant bias (solid red line) was found between the two methods. The limits of agreement (%, ±1.96 standard deviations) are indicated as dashed blue lines.

Total scar size did not differ significantly between the two LGE techniques: mean 5.35 ± 4.32% enhanced volume for dark-blood LGE and 5.24 ± 4.28% for bright-blood LGE, *p* = 0.84. No systematic bias for dark-blood LGE was found in the measurement of myocardial scar size, Figure 3. The LGE methods showed a mean difference of 0.1% (*p* = 0.57), with slightly higher values measured with dark-blood LGE than with bright-blood LGE. The limits of agreement were +1.5% (+1.96 standard deviations) and −1.3% (−1.96 standard deviations).

### 3.4. Reader Agreement and Diagnostic Confidence

Excellent inter-reader agreement was observed for the assessment of the presence of late gadolinium enhancement: κ _dark-blood LGE_ = 0.99 (read 1 vs. 2), 0.97 (read 2 vs. 3), and 0.98 (read 1 vs. 3); and κ _bright-blood LGE_ = 0.98 (read 1 vs. 2), 0.97 (read 2 vs. 3), and 0.97 (read 1 vs. 3). Regarding the confidence level for scar detection, there were no significant differences between dark-blood LGE (3.79 ± 0.41) and bright-blood LGE (3.77 ± 0.46), *p* = 0.60. Regarding the confidence level for the absence of scarring, no significant differences were found between dark-blood LGE (3.88 ± 0.40) and bright-blood LGE (3.91 ± 0.34), *p* = 0.57. When assessing the total quantitative scar size, excellent inter-reader ICC coefficients were observed: 0.93 for dark-blood LGE and 0.94 for bright-blood LGE.

### 3.5. Diagnostic Performance of Dark-Blood LGE

In the dichotomous per-patient assessment of LGE (yes/no), 361 concordant ratings for the presence of LGE in both techniques were given by the readers; and in 661 evaluations, the absence of myocardial scarring was rated concordantly. This adds to a total of 1022/1029 concordant ratings (99%) for the presence or absence of scarring. The numbers of positive and negative LGE counts for both the dark-blood LGE technique and the bright-blood LGE technique are presented in Table 2.

In five cases, one of the readers indicated late gadolinium enhancement (scar) on the dark-blood images but no LGE on the bright-blood images. In two cases, one of the readers detected LGE on the bright-blood images but no LGE on the dark-blood images. All patients with discordant assessments demonstrated only small focal LGE lesions with minimal scar volume (median 1.0% [IQR, 0.6–2.1] for dark-blood LGE, and median 1.6% [IQR, 0.7–2.2] for bright-blood LGE), resulting in low-to-moderate confidence scores for both presence and absence of a scar by all readers.

**Figure 4:**
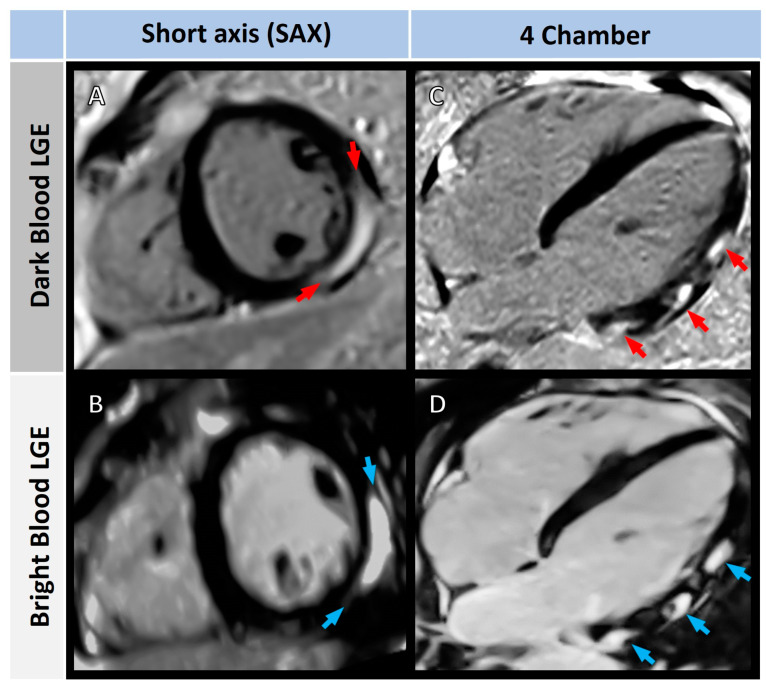
Short-axis dark-blood (top row) and bright-blood (bottom row) images of non-ischemic subepicardial linear LGE in a 19-year-old man with myocarditis. Red and blue arrows indicate the areas of enhancement in (**A**,**B**) short-axis (SAX) views, and in (**C**,**D**) four-chamber views.

Cardiac MRI examples of both LGE techniques displaying typical non-ischemic, subepicardial linear LGE are shown in Figure 4.

Considering conventional bright-blood LGE as the reference standard, dark-blood LGE showed a sensitivity of 99% (confidence interval, CI 98–100), a specificity of 99% (CI 98–100), and an accuracy of 99% (CI 99–100), Table 3. The positive predictive value was 99% (CI 97–99), and the negative predictive value was 100% (CI 99–100). The positive likelihood ratio was 132 (CI 55–317), and the negative likelihood ratio was 0.01 (CI 0.00–0.02). McNemar’s test revealed no significant marginal inhomogeneity between the methods (*p* = 0.45).

## 4. Discussion

This study prospectively evaluated dark-blood late gadolinium enhancement in a head-to-head comparison with conventional bright-blood late gadolinium enhancement imaging for the visualisation of non-ischemic scarring. Our findings suggest that dark-blood late gadolinium enhancement allows for accurate scar detection not only in ischemic scars but also in non-ischemic cardiomyopathies, demonstrating an excellent sensitivity of 99% and an accuracy of 99%.

Accurate detection of scarring by late gadolinium enhancement (LGE) is of paramount importance for cardiac magnetic resonance-based (CMR-based) diagnosis of both non-ischemic and inflammatory cardiomyopathy, as LGE is known to have both high diagnostic and prognostic value. As previously demonstrated, the presence of LGE in non-ischemic cardiomyopathy is associated with a poor prognosis for heart failure and hospitalisation [25]. It portends an increased risk of major adverse cardiac events including sudden cardiac death [8,26]. In addition, not only the presence but also the extent and localisation of LGE areas is predictive. In particular, mid-wall and (antero)septal LGE is associated with increased mortality [27,28]. As a consequence, recent CMR recommendations consider LGE imaging as an indispensable tool for both diagnosis and risk prediction in patients with non-ischemic cardiomyopathies [21,29].

The advantage of dark-blood LGE is optimised to reduce blood pool signal and optimise contrast to the blood pool [30], which may be particularly helpful in cases with possible overlapping or non-specific enhancement patterns on conventional LGE imaging. While the contrast between healthy myocardium and scarring is inherently high in LGE imaging [31], the dark-blood technique is advantageous for detecting small and subendocardial scars without sacrificing contrast or acquisition time [11]. PSIR LGE imaging is generally available on standard CMR scanners from all vendors and scanner types, and the dark-blood-style nulling of the blood pool for PSIR imaging can be easily implemented on any scanner. In addition, dark-blood LGE imaging generally does not require any additional contrast agent or hardware modifications, making it a feasible and cost-effective option for patients with cardiomyopathy.

The proposed technique can be applied on any CMR scanner without requiring costly sequence updates or extensive technical training, as only the inversion time in the PSIR LGE sequence is set differently [17]. Dark-blood LGE imaging can be applied to both 2D and 3D LGE imaging [32].

Dark-blood late gadolinium enhancement has been shown to be more sensitive than conventional bright-blood late gadolinium enhancement in detecting ischemic scarring [12,33]. The improved scar-to-blood contrast improves the delineation of even small subendocardial scars, facilitating the detection of unrecognised myocardial infarction [12]. In this context, the method has recently been histopathologically correlated and confirmed in an animal model with induced myocardial infarction [13]. Unrecognised myocardial infarction is a common finding in patients with coronary artery disease and typically occurs in the posterolateral wall [34]. The question arises whether dark-blood LGE can be used in a standardised comprehensive cardiac MRI protocol to detect ischemic and non-ischemic myocardial scarring and fibrosis, even in equivocal cases. To date, dark-blood LGE has typically been used in cardiac MRI scans dedicated to patients with coronary artery disease and ischemic cardiomyopathy, with no clear recommendation for non-ischemic cardiomyopathy. In general, LGE imaging is optimised to provide contrast between healthy myocardium and focal fibrosis or scarring. However, any LGE technique runs the risk of inadvertently underestimating diffuse fibrosis when there is no healthy myocardium present to serve as a contrast to pathological enhancement [35].

To our knowledge, this is the first study to implement dark-blood late gadolinium enhancement in an exclusively non-ischemic cardiac MRI cohort. The results of our study demonstrate that dark-blood LGE is not inferior to bright-blood LGE in the detection of non-ischemic scarring, which typically spares the sub-endocardium [36]. With an overall concordance of 99% for the presence or absence of scarring by both LGE techniques, dark-blood LGE demonstrated excellent sensitivity, specificity, and accuracy for the detection of non-ischemic scarring in this study. Dark-blood LGE appears to be a reliable diagnostic method for evaluating LGE in both ischemic and non-ischemic cardiomyopathies in daily clinical practice.

As an outlook, dark-blood LGE may become an equivalent acquisition method in LGE imaging for a routine cardiac MRI protocol, considering the proven superiority of dark-blood LGE in the detection of ischemic scars and its non-inferiority in the delineation of non-ischemic scars, as demonstrated in this study. Small undetected myocardial infarction scars or non-ischemic myocardial scars may be better delineated with dark-blood PSIR LGE.

This study has several limitations. First, histologic confirmation of the presence of myocardial scarring was not obtained. Histopathologic correlation has recently been performed in an animal model for ischemic scars [13]; however, it appears difficult to induce non-ischemic scarring in an animal model to allow a direct comparison between LGE technique and histology. Second, data from only one scanner were included, allowing a direct head-to-head comparison between both LGE techniques within the same cardiac MRI examination. Further studies, preferably in a multicentre setting and with an even larger patient population, identical sequence protocols, and scanners from different vendors and different field strengths (1.5 and 3 T), may further investigate the performance of dark-blood LGE (vs. bright-blood LGE) in non-ischemic cardiomyopathies. Third, although the optimal threshold for semi-automated scar quantification of ischemic scarring using dark-blood LGE has recently been investigated using histopathology as a reference standard [37], an optimal threshold for quantification of non-ischemic scarring using dark-blood LGE has not been investigated and is currently unknown [38].

**Key Points:**

1. A prospective single-centre study enrolled patients referred for cardiac MRI for clinical suspicion of non-ischemic cardiomyopathy between January 2020 and March 2023.
2. Dark-blood late gadolinium enhancement showed excellent sensitivity (99%, CI 98–100) and accuracy (99%, CI 99–100) for detecting non-ischemic scarring compared with bright-blood late gadolinium enhancement as the reference standard.
3. Measurements of total scar size did not differ between dark-blood late gadolinium enhancement and bright-blood late gadolinium enhancement.

## 5. Conclusions

Dark-blood LGE has been shown to be non-inferior to conventional bright-blood LGE in the evaluation of non-ischemic scarring, which may have potential implications for future cardiac MRI protocols in the evaluation of unknown cardiomyopathy. As an outlook, dark-blood LGE may become an equivalent acquisition method in LGE imaging for a routine cardiac MRI protocol in the coming years, considering the proven superiority of dark-blood LGE in the detection of ischemic scarring and its non-inferiority in the delineation of non-ischemic scarring, as demonstrated in this study.

## Figures and Tables

**Table 1:** Baseline Characteristics of Study Population (*n* = 343).

Parameter	Study Population
Age (years)	44 ± 17
Age total range (years)	18–82
Female	124/343 (36%)
Male	219/343 (64%)
Final diagnosis by cardiac MRI	
Myocarditis	125/343 (36%)
Non-ischemic cardiomyopathy	109/343 (32%)
Normal	109/343 (32%)

Table summarises the core baseline characteristics of the study population. Values are presented as mean ± standard deviation or numerator/denominator (frequency %).

**Table 2:** Detection of non-ischemic scarring using dark-blood LGE and bright-blood LGE.

		Bright-Blood LGE		
		Positive	Negative	∑
Dark-blood LGE	Positive	361	5	366
	Negative	2	661	663
	∑	363	666	1029

Contingency table depicts dichotomous per-patient evaluations for the presence (positive) or the absence (negative) of enhancement in both the dark-blood technique and the bright-blood technique. *LGE* = late gadolinium enhancement.

**Table 3:** Diagnostic performance of dark-blood late gadolinium enhancement in non-ischemic cardiomyopathy.

	Sensitivity	Specificity	Positive Likelihood Ratio	Negative Likelihood Ratio	Positive Predictive Value	Negative Predictive Value	Accuracy
Dark-blood LGE	99 %	99 %	132	0.01	99 %	100 %	99 %
	(CI 98–100)	(CI 98–100)	(CI 55–317)	(CI 0.00–0.02)	(CI 97–99)	(CI 99–100)	(CI 99–100)

Table depicts the diagnostic test statistics for the detection of non–ischemic scarring by dark-blood late gadolinium enhancement (LGE). Bright-blood LGE was considered as reference standard. *CI* = Confidence interval.

## Data Availability

The datasets analysed in our study are available from the corresponding author on reasonable request.

## Abbreviations

CI = Confidence interval, CMR = Cardiac magnetic resonance, LGE = Late gadolinium enhancement.
